# Re-examination of the standardization of colon cancer surgery

**DOI:** 10.1093/gastro/got020

**Published:** 2013-08-19

**Authors:** Hong-wei Yao, Yin-hua Liu

**Affiliations:** ^1^Department of General Surgery, Peking University Third Hospital, Beijing 100191, China and ^2^Department of General Surgery, Peking University First Hospital, Beijing 100034, China

**Keywords:** colon cancer, surgery, standardization, complete mesocolic excision, D3 lymph node dissection

## Abstract

The standardization of colon cancer surgery has been an area of intense interest. The recent establishment of the complete mesocolic excision (CME) technique has defined the operative approach for colon cancer surgeries and enabled the collection of high-quality oncological specimens for histopathological evaluation. *Standard for the Diagnosis and Treatment of Colorectal Cancer (2010),* issued by the Ministry of Health of China, has provided legal bases for the treatment of colorectal cancers. However, certain confusions remain due to lack of detailed guidelines for operations. This raised the key question: “What is the standardized colon cancer surgery?” The present study re-examined the core ideas of *General Rules for Clinical and Pathological Studies on Cancer of the Colon, Rectum and Anus (seventh edition)* published by the Japanese Society for Cancer of the Colon and Rectum. CME-related studies published in English academic journals between April 2009 and July 2012 were surveyed and analysed. Several technical issues related to the requirement of R0 resection were analysed, including the theoretical basis for the safety range of bowel resection and the rational determination of the range of regional lymph node dissection.

## INTRODUCTION

Based on histology and embryology, the total mesorectal excision (TME) technique has successfully reduced the local recurrence rate of mid-low rectal cancer from 40% to less than 10%. This reduction is achieved through a series of measures including the definition of a ‘holy plane’, recommendation of sharp dissection under direct vision, total mesorectal excision, maintenance of the integrity of the visceral fascia in the specimen, and the pursuit of negative circumferential resection margin [1, 2].

Based on similar theories, in 2009 Hohenberger *et al.* introduced a new technique, termed complete mesocolic excision (CME), for colon cancer [3]. With this new technique, they obtained a 5-year overall local recurrence rate of 4.9% and a reduction of 5-year local recurrence rate from 6.5% to 3.6%. After this study, CME has been regarded as an important contribution to the standardization of colon cancer surgeries. However, our detailed analyses revealed various concerns in respect of their study, ranging from theoretical basis to clinical data.

The present study surveyed CME-related research published between April 2009 and July 2012 in academic journals in English. The objective of this study was to generate objective scientific data for promoting the standardization of colon cancer surgeries.

## ISSUES UNDERLYING CME

CME proposes that a sharp separation of the visceral fascia from the parietal plane can completely isolate the mesocolon, while maintaining the integrity of the visceral fascia, in both anterior and posterior aspects, thereby allowing full exposure and central ligation of the supplying arteries. Theoretically, this procedure maintains the integrity of the embryological envelop around the mesocolon and allows a maximum harvest of lymph nodes. In this respect, CME did undeniably contribute to the standardization of colon cancer surgeries. However, several issues related to the original study on CME require re-examination.

### Range of lymph node dissection

Based on lymphatic anatomy, it has become a consensus that the lymphatic metastasis of colon cancer primarily follows the supplying arteries of colon cancer. Therefore, the determination of the tumor-related supplying arteries is critical for deciding the range of lymph node dissection. In 1995, Toyota *et al.* [4] published *Rationale for extent of lymph node dissection for right colon cancer*, which subsequently became one of the main bases for deciding the range of lymph node dissection in Hohenberger’s study of CME [3]. Toyota *et al.* retrospectively analysed the survival rates and lymph node metastases of 275 patients who underwent D3 lymph node dissection for right colon cancer over a 45-year period (1946–1991). They also reported the prognosis of nine patients with N3 lymph node metastasis and subsequent extended dissections they underwent. The authors concluded that the dissection procedure for right colon cancer involves removal of 10 cm of normal bowel, both proximal and distal to the lesion, and also involves dissection of regional lymph nodes along the main trunk artery up to main nodes. Moreover, infra-pyloric lymph nodes must be dissected when metastasis to the nodes is suspected. In cases of cecal or ascending colon cancer, where the middle colic artery is not the main trunk artery, a right hemicolectomy with resection of only the right branch of the middle colic artery is usually sufficient. Clearly, the study did not recommend extended dissection. In contrast, even in 1995 (the same year as Toyota’s publication), extended lymph node dissection for right colon cancer was reviewed in Japan as only exploratory trials. In fact, the *General Rules for Clinical and Pathological Studies on Cancer of the Colon, Rectum and Anus* (*Japanese Rules*, *first edition*; equivalent English version) published by the Japanese Society for Cancer of the Colon and Rectum (JSCCR) in 1977 [5], already defined rules for grouping of lymph node and their locations, as well as rational performance of lymph node dissection for right colon cancer.

Three decades after the publication of the first edition, the seventh edition of the *Japanese Rules*, published in 2006, adopts essentially the same definition of lymph node grouping and recommends the same range for D3 dissection [6]. In 2010, the Ministry of Health of China issued the *Standard for the Diagnosis and Treatment of Colorectal Cancer* (*Chinese Standard*) [7], which recommends that lymph node dissection for advanced colon cancer without distant metastasis should cover three groups: paracolic lymph nodes, intermediate lymph nodes and nodes at the mesocolic root. Dissection beyond D3 should be considered extended dissection, which still lacks evidence of clinical benefits in patient survival. In comparison, the dissection procedure adopted by Hohenberger *et al.* [3] involved almost full-length skeletonization of the superior mesenteric vessels, which should be considered an extended dissection (i.e. not recommended by the *Japanese Rules* [5, 6]).

### Range of colonic resection

In Hohenberger’s study [3], lymphatic spread of the first group was located in the pericolic lymph nodes, but no more than 8 cm from the primary tumor. Their selection of 8 cm colonic resection, however, lacks data support.

The *Zollinger’s Atlas of Surgical Operations (2011)* [8] provides the following descriptions regarding the range of colonic resection for colon cancer:

- The lymphatic distribution of the large bowel conforms to the vascular supply. Knowledge of this is of great surgical importance, especially in the treatment of malignant neoplasm, because an adequate extirpation of potentially involved lymph nodes requires the sacrifice of a much larger portion of blood supply than would at first seem essential.
- When a curative resection is planned, the tumor and adjacent bowel must be sufficiently mobilized to permit removal of the immediate lymphatic drainage area.
- Extensive block excision of the usual lymphatic drainage areas, combined with excision of a liberal segment of normal-appearing bowel on either side of a malignant lesion, is mandatory.
- Primary anastomosis of the large intestine requires viable intestine, the absence of tension—especially when the bowel becomes distended postoperatively—and a bowel wall of near-normal consistency.

Toyota *et al.* [4] also reported that, in most cases, colon cancer metastasis to the epicolic and paracolic lymph nodes (i.e. Group 1) was within 10 cm from the primary tumor, with only <1% of patients having metastases outside that range.

Therefore, the range of colonic resection should be decided, based on three factors: first, the safety of D1 dissection (instead of direct invasion from the distal and proximal ends of the tumor) requires at least 10 cm resection in both sides; second, the intestinal activity should be considered after high ligation of the supplying arteries (major and minor) in the tumor-related region and subsequent D3 dissection; third, the normal continuity of the intestinal tract should be assured.

### Applicability of CME to cancer more advanced than T3

It is known that total mesorectal excision is more suitable for T1 and T2 tumors than for T3 and T4 tumors. Neo-adjuvant therapy followed by surgery is recommended for T3 and T4 tumors to reduce the incidence of positive circumferential resection margins and local recurrence. As opposed to rectal cancer surgery (i.e. operation in a small pelvic cavity), surgery for colon cancer is performed in a relatively large and open space (i.e. abdominal cavity). Despite this difference, reducing the incidences of positive circumferential resection margins and local recurrence is a common key goal for both surgeries.

In Hohenberger’s study [3], 78% of the subjects had tumors more advanced than T3 (pT3: 63%; pT4: 15%). According to the sixth edition of the TNM staging system, pT3 and pT4 tumors have grown into the outermost layer (i.e. the visceral fascia) of the colon [9]. The visceral fascia has been infiltrated or penetrated by the tumor in reality. Therefore, for tumors in the posterior aspect of the ascending and descending colons (i.e. regions without serosa), it is questionable how to enter the anatomic plane between the visceral fascia and parietal fascia following the CME technique for surgical separation. Moreover, it is also questionable how to achieve R0 resection and ensure negative circumferential resection margin.

### Criteria for cancer staging of subject inclusion

The TNM staging system (sixth and seventh editions) recommends that at least 12 lymph nodes should be examined [9, 10]. When less than 12 lymph nodes were examined, the cancer is rated as Nx (regional lymph nodes cannot be assessed). In Hohenberger’s study [3], the number of examined lymph nodes ranged from 2 to 169 and the authors did not report how subjects with fewer than 12 examined lymph nodes were grouped. Moreover, 0/2 lymph node metastasis cannot be rated as pN0 and 1/6 metastasis cannot be rated as pN1. Therefore, if subjects with <12 examined lymph nodes were not excluded, it would inevitably lead to inconsistent criteria between the experimental group and the control group, thus impairing the comparability of recurrence and survival rates between the two groups.

Additionally, the study included 682 pN0 patients (number of examined lymph nodes: 2–106) and calculated the prognostic relevant cut-off value for the number of examined lymph nodes to be 28 [3]. The difference in cancer-related 5-year survival was statistically significant between the subjects with <28 removed lymph nodes and those with ≥28 removed lymph nodes (90.7 vs 96.3%; *P = *0.018). Similarly, if pN0 was not accurately defined and subjects with <12 examined lymph nodes were not excluded, the comparability between the groups cannot be warranted and therefore the observed differences are questionable.

### General study design and result interpretation

A prerequisite for prospective studies is the selection of research subjects and methods before the actual performance of studies. Hohenberger’s work was a prospective, comparative study using primarily a “standardized surgical approach for colon cancer” [3]. The authors analysed the rates of recurrence and cancer-related survival between three time periods (1978–1984, 1985–1994 and 1995–2002), which represented stepwise changes in the surgical technique and the introduction of a standardized surgical approach. However, since the middle 1990s, first-line chemotherapeutic drugs (oxaliplatin, capecitabine, irinotecan, *et al.*) started to enter clinics and subsequently became widely applied. The appearance of these new chemotherapeutic drugs and regimens has effectively contributed to the overall improvement in the outcome of colon cancer treatment. In Hohenberger’s study, the time period for the construction of the experimental group (1995–2002) coincided with the appearance of these drugs and therapies. In comparison, the time period for the construction of the control group (1978–1984) was reliant on 5-FU as the major chemotherapeutic drug. Therefore, the improvement in outcome recorded in the experimental group may be partially attributable to the benefits of those new drugs and regimens. Hohenberger *et al.*, however, attributed the improvement in the end-point outcomes (i.e. local recurrence and cancer-related survival) solely to the introduction of the CME technique, thus completely ignoring the potential effects of those new drugs and regimens. In our opinion this interpretation is biased and misleading. Additionally, the authors failed to foresee the potential effect of adjuvant chemotherapies and incorporate it as an influencing factor. Consequently, the prospectiveness of that study may be also questionable.

## SURVEY OF CME-RELATED STUDIES

We performed a literature search for CME-related articles published in academic journals in English between May 2009 and July 2012.

Studies conducted in many countries also focused on CME, including both positive and negative opinions. In 2010, West and Hohenberger *et al.* compared CME specimens (from Erlangen, Germany) and ‘standard’ specimens (traditional resection surgery, from Leeds, UK) [11]. They found that compared with the ‘standard’ surgery, CME produced greater distances from the tumor and the closest bowel wall to the high vascular tie, longer lengths of large bowel and ileum removal, a greater area of mesentery resection and a higher lymph node yield. The authors concluded that CME allows the production of superior oncological specimens. In another study, West *et al.* also demonstrated that the quality of specimens can be improved through surgical education [12]. Eihom *et al.* and Bertelsen *et al.* also supported that CME improved the quality of surgical specimens [13, 14]. A recent study compared specimens from CME (performed in Germany) and D3 resection (performed in Japan) [15]. This study found that both techniques showed high mesocolic plane resection rates and long distances between the high tie and the bowel wall. The two techniques also gave similar numbers of positive lymph nodes. However, the CME specimens were significantly longer, resulting in a greater lymph node harvest. Differences in local recurrence and long-term survival between the two groups were not reported but would be of great interest.

Hogan *et al.*, however, argued that CME is merely a new name for the routine operation [16]. They also believed that, without comprehensive analyses of various factors [e.g. differences in surgery (elective or emergency), adjuvant chemotherapies, surgeons and pathologists], it is difficult to determine CME as a dependent contributing factor to patient survival. Importantly, there has been no randomized, prospective study comparing the difference of outcomes between CME and traditional colon cancer surgery, nor systematic prospective studies analysing the application of CME for colonic tumors of different locations, stages, pathological grades and patterns of lymph node metastasis.

### Examination of the Japanese Rules

#### Lymphatic anatomy as the basis for colon cancer surgeries

Since the appearance of the first edition of the *Japanese Rules* in 1977, seven editions have been published in the following decades [6]. The *Rules* considers the grouping of lymph node and radical D3 dissection as the core and standard issues in colon cancer surgery. Based on these, colon cancer surgeries should observe the following basic principles of oncology. The operation should be performed in a ‘no touch’ manner. Before the isolation of a tumor, the supplying artery should be ligated at its root and divided. Isolation should be accurately performed along the right plane between visceral and parietal fascia. The tumor and the surrounding lymph nodes should be removed *en bloc* to maintain the integrity of the bowel and the mesenteric tissues. Regional lymph node dissection should be evidence-based and following standardized procedures. The safety of the distal and proximal resection margins and the circumferential resection margins should be ensured. The operation should strive to achieve R0 dissection.

#### Range of D3 lymph node dissection

The *Japanese Rules* recommends that the lymph node dissection for colon cancer should cover three groups: paracolic, intermediate and central lymph nodes [6]. The *Chinese Standard* specifies that lymph node dissection for colon cancer must cover three groups: paracolic lymph nodes, intermediate lymph nodes and nodes at the root of the mesenteric root [7]. The “nodes at the root of the mesenteric root” in the *Chinese Standard* is equivalent to the “central lymph nodes” in the *Japanese Rules*. A radical D3 lymph node dissection of colon cancer should be performed to remove the paracolic lymph nodes (Group 1), intermediate lymph nodes (Group 2) and central lymph nodes (Group 3) [6].

For colon cancer supplied by the superior mesenteric artery, D1 dissection should remove a certain length of the bowel wall (according to the conditions of vascular supply), usually ≥10 cm from both sides of the tumor. D2 dissection clears lymph nodes along major and minor arteries supplying the tumor. D3 dissection clears lymph nodes distributed near the origins of main arteries related to the tumor blood supply (i.e. ileocolic artery, right colic artery and middle colic artery), which are branching from the superior mesenteric artery.

For colon cancer supplied by the inferior mesenteric artery, D1 dissection covers the similar range to the above. D2 dissection clears lymph nodes along major and minor arteries supplying the tumor. Moreover, if the tumor was located in the sigmoid colon, it should also clear the lymph nodes around the surgical trunk of inferior mesenteric artery. D3 dissection specifically clears the lymph nodes located along the root of inferior mesenteric artery (between the origin of this artery and that of the left colic artery).

#### Issues related to bowel resection

The range of bowel resection distal and proximal to the tumor should be determined, not only by the extent of direct tumor invasion, but also by the requirements for standardized paracolic lymph node dissection (Group 1) and the actual tumor blood supply, so at least 10 cm of bowel on both sides of the tumor should be resected. Furthermore, D3 dissection involves high ligation of supplying arteries originating from the superior and inferior mesenteric arteries (e.g. ileocolic artery, right colic artery, middle colic artery, left colic artery and sigmoid artery), thereby resulting in ischemia of the related bowel sections. Consequently, viability of the bowel sections to be anastomosed is another factor influencing the range of bowel resection. Finally, because the ascending and descending colons are both inter-peritoneal organs and are only partially covered by serosa, appropriately extending the resection ranges of these bowel sections to allow an anastomosis between intra-peritoneal organs may facilitate the restoration of a ‘near-normal consistency’. This ‘selective anastomosis’ is a factor in determining the range of bowel resection and also a potential cause underlying the clinical description of left and right ‘hemicolectomy’.

Therefore, the range of bowel resection should be determined, based on three considerations—standardized paracolic dissection, bowel viability after D3 lymph node dissection and restoration of near-normal consistency after anastomosis—rather than ensuring negative resection margins alone. Any arbitrary extension or reduction of resection range beyond the above considerations is invalid. It should be emphasized that an effective D3 dissection requires a scientific and accurate determination of the major supplying arteries and the lymph nodes in the associated regions. For example, the lymph nodes in the region associated with the middle mesenteric artery are unrelated to cecal cancer and, thus, are not covered by the D3 dissection. No available evidence has supported the benefits of extended vascular ligation (i.e. beyond standardized range), lymph node dissection or bowel resection.

#### Circumferential resection margins

In a resected specimen, the surfaces not covered by peritoneum are referred to as the circumferential resection margins (CRMs). From the perspectives of anatomy and histology, all surfaces of colorectal specimens not covered by serosa (i.e. separated retro- or extra- peritoneally) should be considered as CRMs. By contrast, the colorectal serosal surface itself should not be considered as a CRM. Therefore, instead of only specimens from operations for mid-low rectal cancers, specimens from all colorectal cancer operations involve CRMs [17]. The various sections of the bowel can be classified as intra-peritoneal, inter-peritoneal and extra-peritoneal organs according to their patterns of coverage by the peritoneum. As a result, the ranges of CRMs differ for specimens from different locations. For specimens removed from regions completely covered by serosa (i.e. inter-peritoneal sections), the CRMs include only the resection margins made in the mesentery. For specimens removed from the ascending colon, descending colon and upper rectum (i.e. intra-peritoneal sections), the CRMs include the retro-peritoneal region covered by no serosa. In comparison, all aspects of specimens from the mid-low rectum (i.e. extra-peritoneal section) include CRMs [4]. Therefore, the CRMs in specimens produced by the standardized radical colorectal cancer surgery include the margins made in the covering parietal peritoneum and the root of the mesentery.

Only standardized CME/TME operations involving sharp separation along the holy plane can ensure the integrity of the visceral fascia and negative CRMs. This is a core criterion for evaluating standardized colorectal cancer operations.

For T3 and T4 rectal cancers, neoadjuvant therapies are recommended before surgical resection to reduce the occurrence of positive CRMs. According to the TNM staging system (seventh edition, 2010), T3 and T4 tumors have affected or penetrated the visceral fascia [10]. Therefore, separation following the CME protocol alone would inevitably cause positive CRMs (R1 or R2 resection). As opposed to the rectum (i.e. located in a small pelvic cavity), the colon is located in a relatively large and open space (the peritoneal cavity) and the tumor can be removed by a combined resection, even if it has affected the adjacent tissues or organs. To ensure negative CRMs and R0 resection for such combine resection, the separation plane should be switched from the ‘holy plane’ to another deeper plane, which overtakes the affected adjacent tissues and organs.

## LYMPHATIC ANATOMY-BASED PROSPECTIVE RESEARCH FOR THE STANDARDIZATION OF COLON CANCER SURGERY

In 2012, *CA: A Cancer Journal for Clinicians* published the cancer statistics for the United States population [18]. The 5-year survival rates for stages I-II, III and IV colorectal cancers are 90, 70 and 10%, respectively, indicating a better prognosis with an earlier staging. The TME technique has reduced the high local recurrence of rectal cancer and substantially improved the prognosis of the patients. For colon cancer, which has a relatively low rate of local recurrence, the effects of CME in reducing the local recurrence and improving patient survival need to be further investigated. Therefore comprehensive treatments may be recommended for late-stage colon tumors.

The concept of CME offers viable anatomic layers and operative approaches for standardized colon cancer surgery, as well as higher quality standards for the evaluation of surgical specimens. Thus, the CME technique should be promoted in clinics. The TNM staging system (seventh edition, 2010) provides detailed and quantified N staging and sub-staging criteria [10]. The *Japanese Rules* includes thorough grouping and localization of regional lymph nodes, thus providing an important supplement to the TNM staging system.

A rational combination of the CME technique, TNM staging system, and *Japanese Rules* can ensure a standardized dissection of regional lymph nodes along the ‘holy plane’ according to the pattern of lymph node metastasis and warrant the maximum lymph node harvest. We suggest that prospective studies on the lymphatic metastasis of colon cancer should be performed, based on lymphatic anatomy. These studies may help generate evidence-based guidance for rational practice of radical colectomy and ultimately benefit the patients.

**Conflict of interest:** none declared.
